# The Proper Calculation of Risk Ratios: How and Why

**DOI:** 10.1007/s40614-024-00423-3

**Published:** 2024-10-07

**Authors:** M. Christopher Newland

**Affiliations:** Department of Psychological Science, Auburn University, Auburn, AL USA

**Keywords:** Behavior analysis, Forest plot; Probability, Risk ratio, Relative risk

## Abstract

A recent article (Joslyn, P. R., & Morris, S. L. in *Perspectives on Behavior Science*, 47(1), 167–196, 2024) advocates the use of risk ratios, or relative risk, in behavior analysis. The authors present a strong case for the use of risk ratios and how they might improve the science and application of behavior analysis. Unfortunately, their computation of the risk ratio is incorrect and their examples gloss over important nuances in how risk ratios should be used. The present article corrects the calculations, describes how to determine whether a particular risk ratio differs from a reference group, comments on the importance of stability of the data entering the calculation, and demonstrates approaches to presenting them visually, such as Forest plots.

A recent article published in *Perspectives in Behavior Science* describes the utility of the risk ratio, also called relative risk, in the analysis of behavior (Joslyn & Morris, 2024). Their use can advance quantitative analyses both in the laboratory and in applied settings. The use of risk ratios to quantify behavior change is in the spirit of recent articles published in this and other journals aimed at improving decision making about data collected using single-case experimental designs. Indeed, a special issue of *Perspectives in Behavior Science* was devoted to the application of quantitative techniques in single-case experimental designed (Barnard-Brak et al., 2022).

The risk ratio, properly computed, is an excellent technique with numerous potential applications when the available data are sufficient to support the calculations. Joslyn and Morris’s article is written as a tutorial containing instructions about using the risk ratio and how it might be applied in clinical settings. Unfortunately, the authors define the risk ratio incorrectly, an error that permeates all their worked examples. If perpetuated in the archival literature, the error will introduce confusion about what calculations have been made and what index is being described by authors using this technique. Indeed, Joslyn and Morris’s article already starts this by describing risk ratios using their incorrect calculation side-by-side with ratios using the correct definition. The error will also prevent the application of well-developed statistical theory that has evolved to interpret and compare risk ratios.

## How to Calculate a Risk Ratio

To explain the problem with Joslyn and Morris (2024), consider a contingency table illustrating how a risk ratio can be used clinically (Table 1). A group of individuals is faced with a fatal disease, but an experimental treatment might lower the mortality rate. An “event” in this table would be death from any cause and the letters in the cell refer to the number of individuals dying (“Event”) or not dying (“Nonevent”). The question is whether the mortality rate is higher among those receiving the placebo (A and B) or those receiving a treatment (C and D). Thus, the two mortality rates of interest are the conditional probability of dying given receipt of the placebo, ($\frac{A}{A+B})$ and the conditional probability of dying given receipt of the treatment, ($\frac{C}{C+D}$). The risk ratio (relative risk) is the ratio of these two probabilities:

**Table 1:** Illustration of risk ratios

	Group	
	Placebo	Treatment
Event (E)	A	C
Nonevent (N)	B	D

1 $$RR(Treatment)= \frac{\frac{C}{C+D}}{\frac{A}{A+B}}$$

The relative risk for the treatment group expresses the probability of death among those receiving treatment relative to those receiving the placebo (Cook & Sheikh, 2000; Monaghan et al., 2021). The placebo group is a reference group against which the treatment is compared. This comparison of a single group against a reference group is a simple example but it can be extended to include other variables such as intensity of treatment application, dose, comorbid disorders, or participant demographics.

As is evident in Eq. 1, a risk ratio is a comparison between one group in the numerator against a reference group in the denominator. A risk ratio of 1.0 means that the probability of dying among those in the treatment group is identical to that in the reference group. A risk ratio less than 1.0 means that mortality is less likely in the treatment group (suggesting that the treatment worked) and a risk ratio greater than 1.0 means that the mortality rate is higher in the treatment group (suggesting that the treatment was dangerous).

The risk ratio is a positive number approaching 0 on the left and positive infinity on the right. This creates a skewed distribution because 1.0 is closer to 0 than to infinity on a number line. For calculations and analysis, a log of the risk ratio is computed. The log (risk ratio) can assume values between ± infinity and is centered on 0. The distance to the left of 1.0, as the risk ratio gets smaller, is the same as the distance to the right of 1.0, as the distance gets larger. The log (risk ratio) is often distributed normally, which simplifies other calculations. If inspection reveals a nonnormal distribution, then transforms or bootstrapping might be warranted, but those topics are beyond the scope of this article.

The definition of the risk ratio in Eq. 1 is the one used in the statistical and methodological sources cited by Joslyn and Morris (2024), such as the National Cancer Institute (2024) and the Stat Pearls web page sponsored by the National Library of Medicine (Teeney & Hoffman, 2023). It is also the one used by many other citations in their article and is the definition found in other tutorials about this approach (Cook & Sheikh, 2000; Monaghan et al., 2021). Joslyn and Morris, however, define the risk ratio differently. They assert that “[r]isk ratios can be calculated by dividing the probability... of behavior given some variable by the *overall* probability... of that behavior.” (p. 169; emphasis added). As they describe it, the risk ratio is the conditional probability of, for example, dying given treatment divided by the unconditional probability of dying in the entire population sampled. No specific information about a reference (or control) condition is incorporated into their calculation and, it is important to note, there is no mention of the specific probability of *not* dying. There is also no mention of taking the logarithm of the risk ratio, which is important for many reasons as described below. Referring to Table 1, Josylin and Morris’s “risk ratio” is calculated as:

2 $$RR\left(Treatment:err\right)= \frac{\frac{C}{C+D}}{\frac{A+ C}{A+B+ C+D}}$$

Equation 2 is problematic for a number of reasons, of which I will mention two. The first, and most obvious, is that the calculation is different than every calculation of risk ratio in the published literature with some recent exceptions involving one of the article’s authors (Morris & Vollmer, 2022a, b; Perez et al., 2021).^1^ Joslyn and Morris argue that the risk ratio should be calculated differently for behavioral phenomena, but their rationale is not made explicit. There is no reason that a numerical calculation should depend on the discipline for which it is calculated. It would be highly confusing if risk ratios as defined in medicine, epidemiology, substance use disorders, and applied behavior analysis were all calculated differently.

A more subtle problem is that the numerator and denominator are not independent in Eq. 2. In Eq. 1, a death in the treatment group does not affect the risk calculation in the placebo group (the denominator) and vice versa. In Eq. 2, however, a death in the treatment group also affects the overall mortality in the denominator, so the numerator and denominator covary. Thus, the two comparison groups are intertwined, not independent. This issue becomes important when using the risk ratio to determine the impact of an intervention or whether some condition affects the risk of, in the above example, death.

To illustrate the difference in the calculations, consider Demonstration Number 2 from Joslyn and Morris (2024) describing the number of 10-s intervals in which participants covered their ears as a function of the loudness of ambient auditory stimuli. Table 2 below shows ear-plugging by one of the participants, Harold. This behavior presumably is maintained because it reduces the aversiveness of a loud stimulus. Table 2 reconstructs their calculations of the risk ratio (contained in their Table 2) and adds proper calculations of risk ratios for comparison. Column 1 shows the ambient loudness in decibels (dB). Column 2 shows the number of intervals available for consideration. There were 4249 intervals at < 80 dB, 2012 at 80–89 dB and so on. The number of intervals at specific loudness levels sums to 7,200. Column 3 shows the number of intervals in which ear-plugging occurred. Column 4 shows their “unconditional probability,” the total number of intervals with ear-plugging divided by 7,200. The conditional probabilities are shown in Column 5. This is the number of intervals with ear-plugging at a particular noise level divided by the number of intervals at that noise level. For example, at < 80 dB the conditional probability is 849/4240 = 0.20. Column 6 shows the erroneous risk ratio as calculated in Joslyn and Morris (2024). This is the conditional probability divided by the unconditional probability (0.20/0.30 = 0.67 for < 80 dB). The erroneous risk ratio for the 80–89 dB condition is 1.3 (0.4/0.3), and so on.

**Table 2:** Ear-Plugging by Harold

Stimuli	Number of Intervals	Intervals with Ear Plugging	Unconditional Probability	Conditional Probability	Risk Ratio (erroneous)	Risk Ratio (Correct)
All	7,200	2,183	0.30			
< 80 dB	4,249	849		0.20	0.67	1.0
80–89 dB	2012	795		0.40	1.30	2.0
90–99 dB	915	523		0.57	1.89	2.9
>99 dB	24	16		0.67	2.20	3.3

Taken from Table 2 from Joslyn and Morris (2024)

The correct risk ratio (rightmost column) uses the lowest auditory stimulus condition (< 80 dB) as the reference group and compares all the others against that standard. It does not use their unconditional probability in any calculation, so that column should not be included in any tables showing risk ratios. As ambient loudness increases, the probability (risk) that Harold plugs his ears increases 2-, 2.9-, and 3.3-fold as compared with the reference group. All the comparisons in the rightmost column are against a single reference group and all are higher than the incorrect risk ratio. Are they independent? They are independent if a change in the number of intervals with ear-plugging in the comparison condition (or any other condition) would not affect the number of intervals with ear-plugging in the other conditions. This is implied in the description in Joslyn and Morris’s (2024) Demonstration Number 2, but it is difficult to ascertain with certainty.

It might be noted that the risk ratio is different from an odds ratio, a related calculation often used in similar situations. Their distinctions are important but, to keep this article brief, they will not be discussed in detail here. That said, one difference worth noting is that the odds are the number of events divided by the number of nonevents whereas the risk is the number of events divided by the number of events *plus* the number of nonevents (Monaghan et al., 2021). From Table 2, the odds of ear-plugging for the < 80 dB is 849/(4249—849), the number of intervals during which the ears were covered divided by the number of intervals during which they were not covered. Thus, the odds tell us that an event is X times more likely than a nonevent whereas the risk tells us the probability of an event occurring in one population.

## Why Calculate It Properly?

The first reason for calculating risk ratios, or relative risk, as in Eq. 1 is that everybody else does it this way. When the term is used appropriately then it is understood correctly. Confusion ensues when usage is idiosyncratic. This is exemplified in Joslyn and Morris’s (2024) discussion of the utility of risk ratio, where (pp. 160–169) where they blur the distinction between the two calculations by citing articles that use the term correctly (e.g., Hackshaw et al., 2018) and those that do not (e.g., Morris & Vollmer, 2022a, b) without noting the difference.

A second reason is that Eq. 2 guarantees that the numerator and denominator are not independent of one another. The lack of independence becomes especially problematic if we wish to know if the probability of dying among those receiving treatment likely differs from that of those receiving a placebo. We would like to know, for example, if a risk ratio of 2.0 can be distinguished from 1.0, or if that value simply reflects normal variability. What if the risk ratios were closer to 1.0: 1.5, 1.01, or 0.8? Are these likely to be different? Determining whether two risk ratios are different is difficult, if not impossible, by visual examination alone because both sample size and the magnitude of the difference between two groups must be considered. Quantifying that judgment entails comparing independent groups; it cannot be accomplished meaningfully when the numerator and denominator covary.

## How to Compare Two or More Conditions

How to compare conditions is not addressed in Joslyn and Morris (2024), but it is crucial when interpreting risk ratios. The proper calculation of risk ratio allows the investigator to apply theoretically informed statistical approaches, such as the calculation of confidence intervals, to compare groups. A common approach to comparing risk ratios is to calculate the confidence interval surrounding the risk ratio, an approach that is well-grounded in theory. There are numerous sources for calculating confidence intervals for risk ratios, including online tutorials (Boston University School of Public Health, 2024), web-based calculators, or software such as R or SAS. A particularly simple, though not intuitive, calculation that is easy to incorporate into a spreadsheet is provided in Morris and Gardner (1988).

The confidence interval describes the range of values in which a measure, such as a risk ratio, might fall if the study were conducted multiple times under the same conditions with the same population. If a 95% confidence interval includes the value of 1.0, indicating that the risk for a condition is identical to the reference condition, then the risk is viewed as indistinguishable from the reference condition and is considered nonsignificant. If 1.0 is outside the 95% confidence intervals then the risk is considered to be different from the reference group. The size of the confidence interval used, 90%, 95%, or 99%, for example, may depend on the application (Hazra, 2017).

**Fig. 1:**
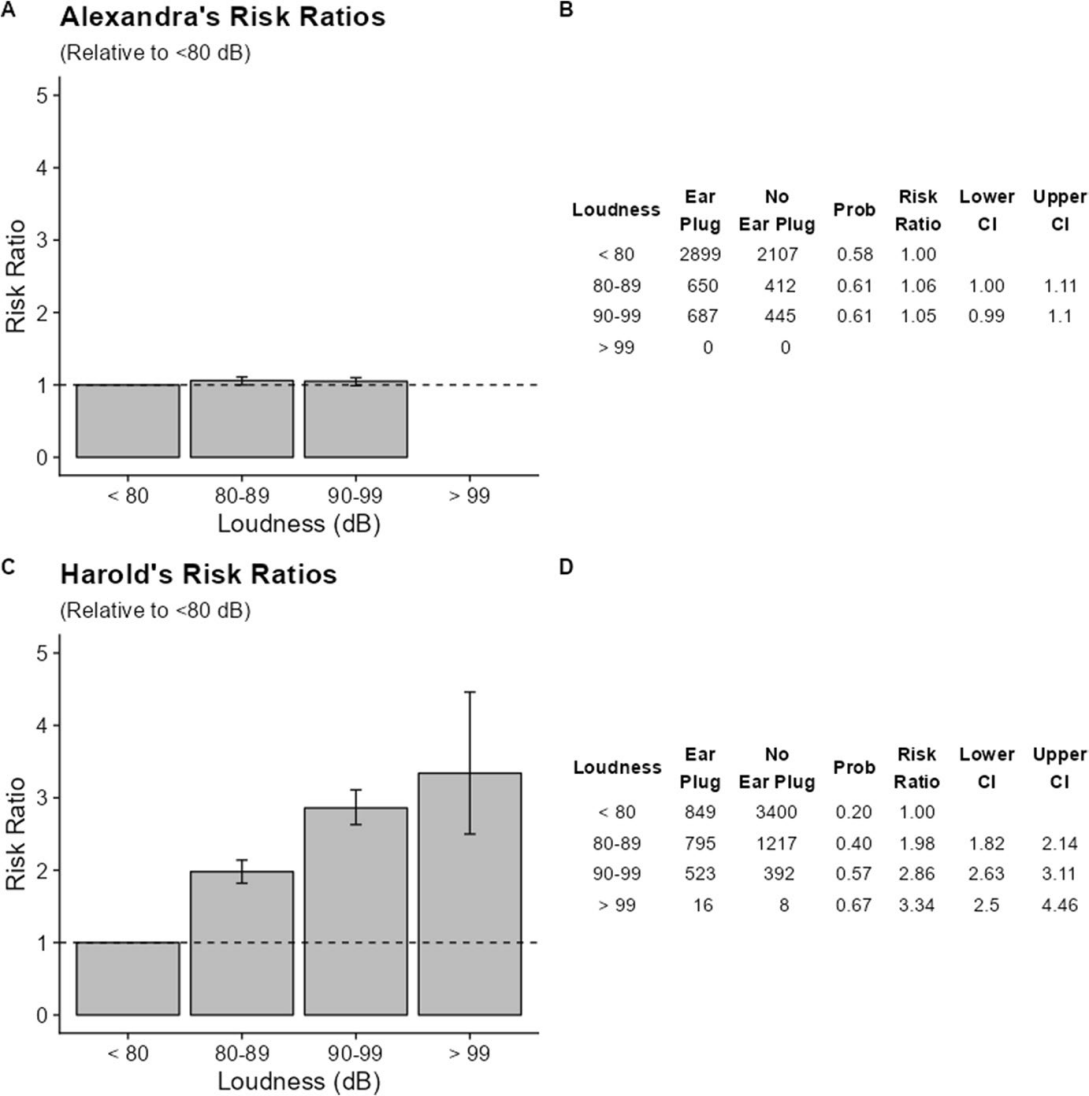
Risk Ratios and Their 95% Confidence Intervals Describing the Voluntary Use of Ear Plugs for Two Participants. Data from Joslyn and Morris (2024)

The study of ear-plugging in Table 2 will be used to exemplify how 95% confidence intervals guide comparisons of different conditions with a reference condition. Here, we use both individuals described in that demonstration, Alexandra and Harold. Figure 1A and C plot the risk ratio for two participants as bar graphs with error bars showing 95% confidence intervals. All conditions are compared against their reference condition, < 80 dB, which is shown as the leftmost bar. A risk ratio of 1.0, indicated by a horizontal dashed line, means that the risk in that condition is identical to the reference condition. The risk ratio for the reference condition is always 1.0. For Alexandra, the probability of ear-plugging was very close to that of the reference condition for the 80–89 dB and 90–99 DB conditions. Alexandra never experienced a noise condition > 99 dB.

The tables to the right of the bar graphs in Fig. 1(B, C) show the number of intervals with and without ear-plugging, the probability of earplugging (intervals with ear-plugging divided by intervals with and without ear-plugging), the risk ratio and the lower and upper confidence intervals of ear-plugging. For Alexandra (Fig. 1A and C), the risk ratios are close to 1.0 for the two conditions that Alexandra experienced. It is uncertain whether the lower 95% confidence interval for the 80–89 dB condition includes 1.0. It would require more decimal points to determine that with certainty but doing so would not change the ambiguity of the decision: if it does not include 1.0 it is only by rounding error. The confidence intervals for the 90–99 dB condition do include 1.0 because the lower one, 0.99 is less than 1.0 and the upper one, 1.01, is greater than 1.0. If one takes the perspective that the confidence interval is a bright line then only the 80–89 dB condition would be considered different from the reference and the other one would be ambiguous. However, the ratios are close to 1.0 and there is no clear relation to noise magnitude, so the investigator or clinician would be justified in questioning the relation between ear-plugging and ambient noise. In contrast, Harold’s risk ratios (Fig. 1C and D) increased systematically with noise and all three conditions were clearly greater than 1.0. None of the three 95% confidence intervals included 1.0 in their span so they are distinguishable from the reference. It is clear that Harold’s ear-plugging was influenced by ambient sound.

A second study described by Joslyn and Morris (2024) will be used to illustrate what kind of data are, and are not, suitable for calculating risk ratios and to demonstrate a concise and visually appealing approach that is often used to display risk information: Forest plots. The study is described in Joslyn and Morris (2024) as “Demonstration 1: Demonstrating High- and Low-Risk Days of the Week.” In brief, aggressive incidents among 120 juvenile offenders who resided in a secure juvenile detention facility were recorded for a year. The question was whether aggressive incidents varied across days of the week.

To answer their question, the authors divided the total number of aggressive events on, for example, a Monday by the number of Mondays in that year. An attempt to place this information in the 2 X 2 table (Table 1) reveals why this calculation is not suitable for a risk ratio or for answering the very question posed in the title of the demonstration. To fill in Table 1, it is necessary to have information about both the number of events that occur in a specific time period and the number of nonevents in that same period. The former is the number of Mondays with an aggressive event. The latter, the number of nonevents, is the number of Mondays without an aggressive event. Simply dividing the total number of events across all Mondays by the number of Mondays does not reveal how many Mondays occurred with aggression and without aggression. Some Mondays may pass with no such event, some with only one, and some with many. In fact, to take an extreme example, it is possible that all aggressive events occurred on only 1 or 2 Mondays and 50 or 51 Mondays had none. Their calculation hides such important information.

**Fig. 2:**
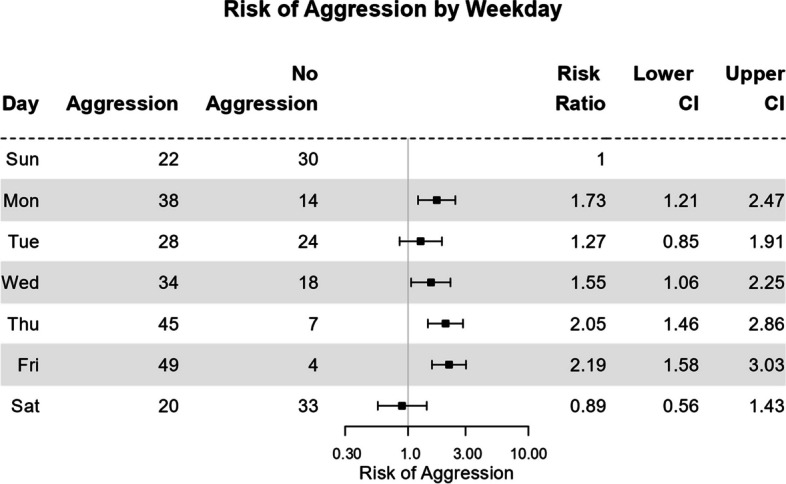
Risk of Aggression across Days of the Week. The horizontal axis is scaled logarithmically. Error bars show 95% confidence intervals of the risk ratio. Data, obtained from Joslyn and Morris (2024), are modified to serve as an example (see text)

The dataset will be modified by pretending that the number of aggressive events reported across all Mondays actually describes the number of Mondays on which an aggressive event occurred. This is not what was reported but it furthers the example here. The illustrative results are shown in Fig. 2. For example, the original article reports 38 aggressive incidents occurred on a Monday during the observation. Figure 2 incorporates the assumption that there were 38 Mondays with an aggressive event (Fig. 2, Column 2) and therefore 52 minus 38 Mondays without one. Figure 2 shows the number of days with and without aggression broken out by weekday. For example, there were 22 Sundays with an aggression event and 30 Sundays without one. Other details can be found in Joslyn and Morris (2024).

Figure 2 shows an example of a Forest plot, a format that is commonly used to illustrate both risk ratios and odds ratios (Dettori et al., 2021). As is often, but not always, done with such a plot, the contingency table accompanies the Forest plot. The top row is the reference group, Sunday (selected arbitrarily). All risk ratios are compared against that reference. The graph shows the risk ratio for Monday through Saturday, so it is the risk of aggression on, say, Monday (38/(38 + 14) divided by the risk of aggression on Sunday (22/(22 + 30)) = 1.73. Next, the probability of aggression on Tuesdays is compared against Sunday, and so on through Saturday. Error bars show the 95% confidence intervals of the risk ratios. The actual ratios are on the right and confidence intervals. No risk ratio is plotted for Sunday (by convention) because that value is defined as 1.0.

Recall that if an error bar includes 1.0 (indicated by the vertical gray line) then the risk is considered to be indistinguishable from the reference condition, Sunday. So, the risk of aggression on Monday is 1.73 times that on Sundays but that can range from 1.21 to 2.47. Monday has a higher risk of aggression than Sunday. For Tuesday, the lower and upper confidence intervals are 0.85 and 1.9; the no-effect risk of 1.0 is included in that span so Tuesdays are indistinguishable from Sunday. Overall, the Forest plot shows that the risk of aggression is usually higher on weekdays (Tuesday being the exception) than the weekend.

The upper and lower confidence intervals are positioned asymmetrically around the mean. For example, for Monday, the lower one is 1.73—1.21 = 0.52 units from the mean but the upper one is 2.47—1.73 = 0.74 units from the mean. This asymmetry arises because the mean and confidence intervals are calculated using log ratio) and then converted back to actual ratios for ease of interpretation. They look symmetric in Fig. 2 because the horizontal axis is scaled logarithmically (the number reflects the actual, non-log-transformed risk ratio).

## Stationarity

**Fig. 3:**
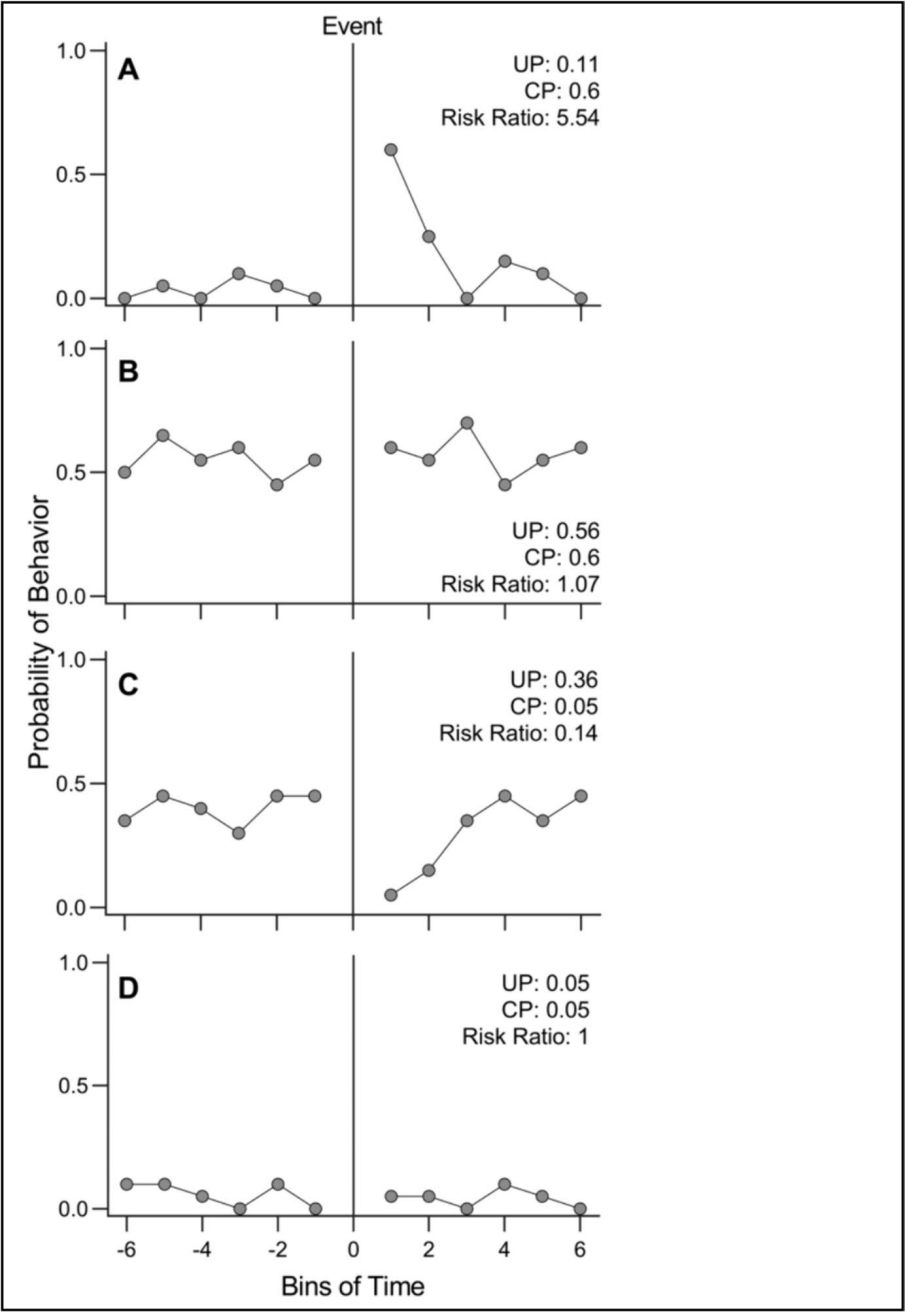
Hypothetical Data to Illustrate the Use of Risk Ratios. The risk ratios are calculated incorrectly and inappropriately include data from a transient phase (see text). From Joslyn and Morris (2024) with permission

One concern when comparing two or more conditions is the stability, or stationarity, of the data. The risk ratio examines the risk, or probability, of an event occurring in one condition as compared with a second condition. Making that comparison assumes that the behavior in both conditions is stable and applying that comparison assumes, sometimes implicitly, that the context to which it is applied is also stable. This requirement means that applying a risk ratio to examine a behavior before (the “A” phase) and after (the “B” phase) an intervention in a multiple baseline must be performed only with behavior that is stable. The problem that can arise is illustrated in Fig. 3 (reproduced from Joslyn and Morris’s (2024) Fig. 1) in which putative risk ratios are calculated before and after an intervention. This figure shows hypothetical data to illustrate how to calculate a risk ratio in an A-B experimental design, though it could easily be extrapolated to more complex designs. An intervention occurs at bin 0 (vertical line) so a comparison is made between the session after the intervention (positive bins) against sessions before the intervention (negative bins).

In all four cases the risk ratios are calculated improperly by using Eq. 2. In two cases (Panels A and C), the data entering the calculation includes behavior that is clearly transient so the result is misleading. To calculate an interpretable risk ratio, it is necessary to identify the beginning of steady state during the B phase and compare it with the (presumable) steady state at the end of the A phase. Based on a visual assessment of when behavior in the intervention phase stabilizes, calculation of a risk ratio should omit at least the first two data points after the intervention for Panel A and the first three data points after the intervention for Panel C.

## Concluding Remarks

The article by Roslyn and Morris describes a powerful method for characterizing behavior under different environmental conditions and quantifying behavior change. A large literature describing the interpretation and presentation of risk ratios, and their utility in different disciplines, can be drawn upon by those interested in applying them in behavior science if the risk ratio/relative risk is computed correctly, The difference between the Joslyn and Morris (2024) definition of risk ratio (Eq. 2), and the one used in the rest of the scientific literature (Eq. 1), may appear subtle but it is actually quite significant. Two important consequences that could arise from their improper use are described here. One is confusion that arises if the term “risk ratio” used idiosyncratically in behavior analysis. This is important for their proper use in practice, and in attempts to review a scientific or clinical literature. Those conducting narrative reviews or meta-analyses must have confidence that risk ratios are computed correctly. A risk ratio computed incorrectly cannot be incorporated into reviews or meta-analytic summaries without distorting the conclusions. Groups such as the What Works Clearinghouse (2024) may even omit articles that have incorrect or nonstandard calculations. The second consequence is that behavior analysts miss out on a significant and long-standing literature that can be drawn upon to improve the analysis of behavior. Both of these consequences would represent loss to our science and practice.

## Data Availability

The author confirms that all data generated or analyzed for this article are contained in the article or in the literature cited.

## Footnotes

1 Joslyn and Morris also include the citation (Morris & Vollmer, 2020a) as involving risk ratios but that article does not do so. A different article published in 2020 does use risk ratios, however (Morris & Vollmer, 2020b).
